# Heterogeneity of thromboembolic risk across ALK inhibitors: a retrospective real-world study identifying crizotinib-specific signals and high-risk subpopulations

**DOI:** 10.3389/fphar.2026.1855116

**Published:** 2026-09-07

**Authors:** Rong Hu, Qing Ai, Hui Zhang, Min Liu, Yuan Qiu, Yingyi He

**Affiliations:** 1 Department of Pharmacy, Fujian Children's Hospital (Fujian Branch of Shanghai Children's Medical Center), College of Clinical Medicine for Obstetrics & Gynecology and Pediatrics, Fuzhou, China; 2 Department of Hematology/Oncology, Fujian Children's Hospital (Fujian Branch of Shanghai Children's Medical Center), College of Clinical Medicine for Obstetrics & Gynecology and Pediatrics, Fuzhou, China; 3 Department of Thoracic Surgery and Oncology, The First Affiliated Hospital of Guangzhou Medical University, State Key Laboratory of Respiratory Disease, National Clinical Research Center for Respiratory Disease, Guangzhou Institute of Respiratory Health, Guangzhou, China; 4 Department of Pediatrics, The Chinese University of Hong Kong, Hong Kong SAR, China; 5 State Key Laboratory of Cellular Stress Biology, School of Life Sciences, Faculty of Medicine and Life Sciences, Xiamen University, Xiamen, China

**Keywords:** anaplastic lymphoma kinase inhibitor, FAERS, AMES, NSCLC, pharmacovigilance, real-world, thromboembolic event

## Abstract

**Background:**

Patients with anaplastic lymphoma kinase (ALK)-positive malignancies increasingly rely on ALK inhibitors (ALKis) as first-line therapy, yet thromboembolism (TE) across the ALKi family and its impact on patients’ safety remain incompletely characterized, particularly in the real-world populations.

**Objective:**

The aim of this study was to evaluate the real-world TE risks associated with ALKis using pharmacovigilance data mining and to identify and characterize the high-risk contributors to TE.

**Methods:**

We extracted 52,808 adverse event (AE) reports from the FDA Adverse Event Reporting System (2013–2024), including 17,124 ALKi-treated patients. TE signal strength was calculated via disproportionality analysis and presented by the lower limit of reporting odds ratio (ROR_025_) and information component (IC_025_). Multivariate logistic regression and time-to-onset (TTO) analyses were conducted to characterize the risk factors.

**Results:**

Among ALKi users, 251 TE cases were identified. Crizotinib exhibited significant TE safety signals for embolism (ROR_025_ = 2.66; IC0_25_ = 1.19), pulmonary artery thrombosis (ROR_025_ = 4.94; IC0_25_ = 1.01), pulmonary thrombosis (ROR_025_ = 1.29; IC0_25_ = 0.13), and venous thrombosis limb (ROR_025_ = 1.66; IC0_25_ = 0.08). Older age (OR = 1.006, P = 2.40e-06), male sex (OR = 1.47, P = 2.00e-16), and crizotinib use (OR = 2.79, P = 1.59e-05) independently predicted fatal TE outcomes. Female patients <65 years experienced earlier TE onset (median 14 vs. 44–53 days; P = 0.006).

**Conclusion:**

This study validates the elevated TE safety signal strength of crizotinib in the real-world settings, while no disproportionate TE signals were detected for other ALKi in the FAERS database. Clinicians could prioritize agent-specific risks, especially in older male patients and during early treatment phases.

## Introduction

Anaplastic lymphoma kinase (ALK) inhibitors (ALKis) have become the cornerstone of first-line therapy for ALK-positive malignancies, with ongoing debates focusing on the optimal agent choice. For surgical candidates with ALK-positive lung cancer, the perioperative management of ALKis remains challenging due to potential thromboembolic complications that may increase postoperative morbidity. Current guidelines lack evidence-based recommendations on ALKi-specific TE risk stratification during surgery windows. Evidence indicates that next-generation ALKis provide longer progression-free survival compared to crizotinib (Damuzzo et al., 2025) in non-small cell lung cancer (NSCLC), yet the drug adverse events (AEs), particularly regarding rare but severe AE (SAEs), remain less clearly defined in therapeutic regimen design and warrant in-depth investigation.

Patients with ALK-rearranged NSCLC exhibit a notably elevated risk of thromboembolism (TE) compared to other molecular subtypes (Zer et al., 2017; Roopkumar et al., 2021), and TE is associated with poorer survival outcomes across diverse lung cancer populations (Roopkumar et al., 2021; Mahajan et al., 2022). Beyond the pathophysiological predispositions, ALK-targeted therapies also contribute to an increased risk of TE (Qi et al., 2024), and TE is observed in both adult (Zaborowska-Szmit et al., 2024) and pediatric (Lowe et al., 2023) ALK-positive malignant patients. A recent systematic review and Bayesian network meta-analysis of eight clinical trials highlighted an elevated risk of TE with crizotinib relative to other ALKis and chemotherapy (Qi et al., 2024). However, these clinical trial-based findings are derived from highly selected populations under controlled conditions, and it remains unknown whether they are generalizable to the heterogeneous real-world setting, where patients present with varying comorbidities, and potential off-label usage. Furthermore, real-world prescription data from Greece (2020–2022) have documented a marked transition in clinical practice that crizotinib utilization among ALK-positive NSCLC patients decreased from 60% to 16%, while alectinib increased to 59% over the same period (Gourzoulidis et al., 2025). This rapid adoption of the next-generation ALKi underscores the contemporary relevance of characterizing drug-specific TE safety profiles, even for agents with decreasing use, as patients previously exposed to crizotinib may still carry legacy risks, and resource-constrained settings may continue to rely on this agent.

Therefore, the primary aim of this study is to explore whether the heterogeneous TE risk across individual ALK inhibitors, particularly the crizotinib-specific signal, remains robust and detectable in the real-world pharmacovigilance database. FDA Adverse Events Reporting System (FAERS), now known as The FDA's Adverse Event Monitoring System (AEMS), is a publicly available real-world big data repository collecting world-wide drug safety reports submitted by healthcare professionals, patients, and pharmaceutical companies (U. S. Food and Drug Administraction, 2026). Through detailed analysis of post-marketing safety reports in FAERS, we could quantify and compare the independent TE risk for each commercially available ALKi, thereby providing real-world validation of the prior meta-analytic evidence. Following this, we also aimed to characterize the TE patterns and to identify high-risk factors and the impact on patients’ outcomes, thus informing clinicians on balancing efficacy with safety considerations when selecting ALK-targeted treatments for ALK-positive malignant patients. This retrospective disproportionality study has been reported in line with the READUS-PV guideline (Fusaroli et al., 2024), and no artificial intelligence has been applied in this research.

## Methods

### Ethics statement

The FAERS database is publicly accessible, and de-identify and anonymize patient records. Consequently, ethical clearance and informed consent are exempted for this study.

### Data source and processing

Pharmacovigilance data mining was conducted as described previously (Shen et al., 2024). TE events were extracted from malignant patients’ AE reports deposited in the FAERS database. Malignant diagnoses were based on the reported indication of drug use, aligned with the preferred term (PT) from the Medical Dictionary for Regulatory Activities (MedDRA) version 25.1. Five commercially available ALKi, namely, crizotinib, alectinib, brigatinib, ceritinib, and lorlatinib, were included in this study, and the inclusion time period was set from 2013 semester 1 to 2024 semester 4. As ensartinib was only recently approved by the FDA on 18 December 2024, it was excluded from this analysis. A two-step data cleaning procedure was conducted to directly remove the duplicated reports, followed by removing the highly suspected duplication with all the same values in “Sex,” “Age,” “Country,” “Event Date,” “Adverse Reaction,” “Drug,” and “Indication” as described (Shen et al., 2024).

### Pharmacovigilance signal mining

Retrospective disproportionality analysis is the primarily employed methodology to assess the potential AE risk in a pharmacovigilance study (Fusaroli et al., 2024), and reporting odds ratio (ROR) and information component (IC) are the two major parameters widely adopted to examine the likelihood of an AE for a specific drug. As described previously (Hu et al., 2025), ROR and IC, along with their lower limits (ROR_025_ and IC_025_), were calculated. In this study, “a” and “b” denote the number of TE and other AE reports in ALKi users, respectively, while “c” and “d” represent the number of TE and other AE cases without ALKi therapy, respectively. The formulae are presented in Equations 1–4:

$$\text{ROR}=\frac{a\;*\;d}{c\;*\;b},$$ (1)

$${\text{ROR}}_{025}=e^{\ln\left(\text{ROR}\right)-1.96\sqrt{\frac{1}{a\;}+\frac{1}{b\;}+\frac{1}{c\;}+\frac{1}{d}}},$$ (2)

$$\text{IC}=\log_{2}\frac{a+0.5}{\frac{\left(a+b\right)\left(a+c\right)}{\left(a+b+c+d\right)}+0.5},$$ (3)

$${\text{IC}}_{025}=\text{IC}-3.3*\frac{1}{\sqrt{a+0.5}}-2*\frac{1}{\sqrt{{\left(a+0.5\right)}^{3}}}.$$ (4)

A positive AE signal is defined as “a” ≥ 3, with ROR_025_ > 1 or IC_025_ > 0. In this study, if a PT met these criteria for both ROR_025_ and IC_025_, it would be graded as a high-potential TE signal warranting further attention. Unlike a purely exploratory signal detection study, our analyses were conducted in a hypothesis-driven, confirmatory manner. ROR and IC estimates presented here are not intended to quantify comparative incidences or causal effect sizes between inhibitors. Rather, they serve as signal substantiation, indicating the presence or absence of a statistically disproportionate reporting pattern in the real-world setting. The absence of positive signals should be interpreted as “no detectable disproportionate signal” rather than “confirmed lower TE risk” from the pharmacoepidemiological perspective.

To address the etiological heterogeneity between arterial and venous thrombosis, all identified TE-related PTs were also classified into three predefined categories based on anatomical attribution: venous TE (VTE), arterial TE (ATE), and unspecified TE for PTs lacking explicit vascular attribution (e.g., embolism and thrombosis). Given the extremely low event counts in the ATE category (n = 5) and the substantial proportion of unspecified terms (31.4%) extracted from FAERS, quantitative disproportionality analyses (ROR and IC) for pooled subgroup estimates were not performed to avoid statistically unstable results. Instead, individual PT-level signals for all terms with case counts ≥3 were transparently presented to preserve data granularity.

### Multivariate logistic regression and time-to-onset (TTO) analyses

Multivariate logistic regression was performed to identify independent predictors of fatal outcomes among patients who experienced TE. The outcome variable was fatal outcome (binary; fatal vs. non-fatal). Available covariates extracted from FAERS included ALKi subtype, sex, and age (continuous variable). Only cases with complete data for all covariates were included in the final model. Odds ratios (ORs) and 95% confidence intervals (CIs) were calculated. The forest plot was generated to display the ORs and their corresponding 95% CIs for all covariates included in the final model.

Only patients with a documented time to onset (TTO) within the first year of ALKi therapy were included in the TTO analysis to minimize confounding from long-term follow-up heterogeneity. Subgroup analyses of TTO were performed, stratified by sex (male vs. female) and age group (<65 vs. ≥ 65 years), to explore potential demographic modifiers of early TE occurrence. Differences were compared using the log-rank test, and cumulative incidence curves were generated using the Kaplan–Meier method. To account for multiple comparisons across the four subgroups, the P-values from pairwise log-rank tests were adjusted using the Benjamini–Hochberg method.

### Statistical analyses

Histograms, line charts, and heat maps were plotted to visualize the chronological and geographical distributions of all the AE and TE reports, as well as the annual proportion of TE reports following ALKi therapy. A Sankey diagram was drawn to present the trajectory path between diseases, ALKi types, TEs, and outcomes. The Mann–Whitney U test and the Kruskal–Wallis test were used to compare the median time to TE onset between ALKi users and non-users, as well as among groups stratified by sex and age. All statistical analyses were performed using R (https://www.r-project.org/; version 4.5.0). A two-sided P-value less than 0.05 is considered statistically significant.

## Results

**FIGURE 1:**
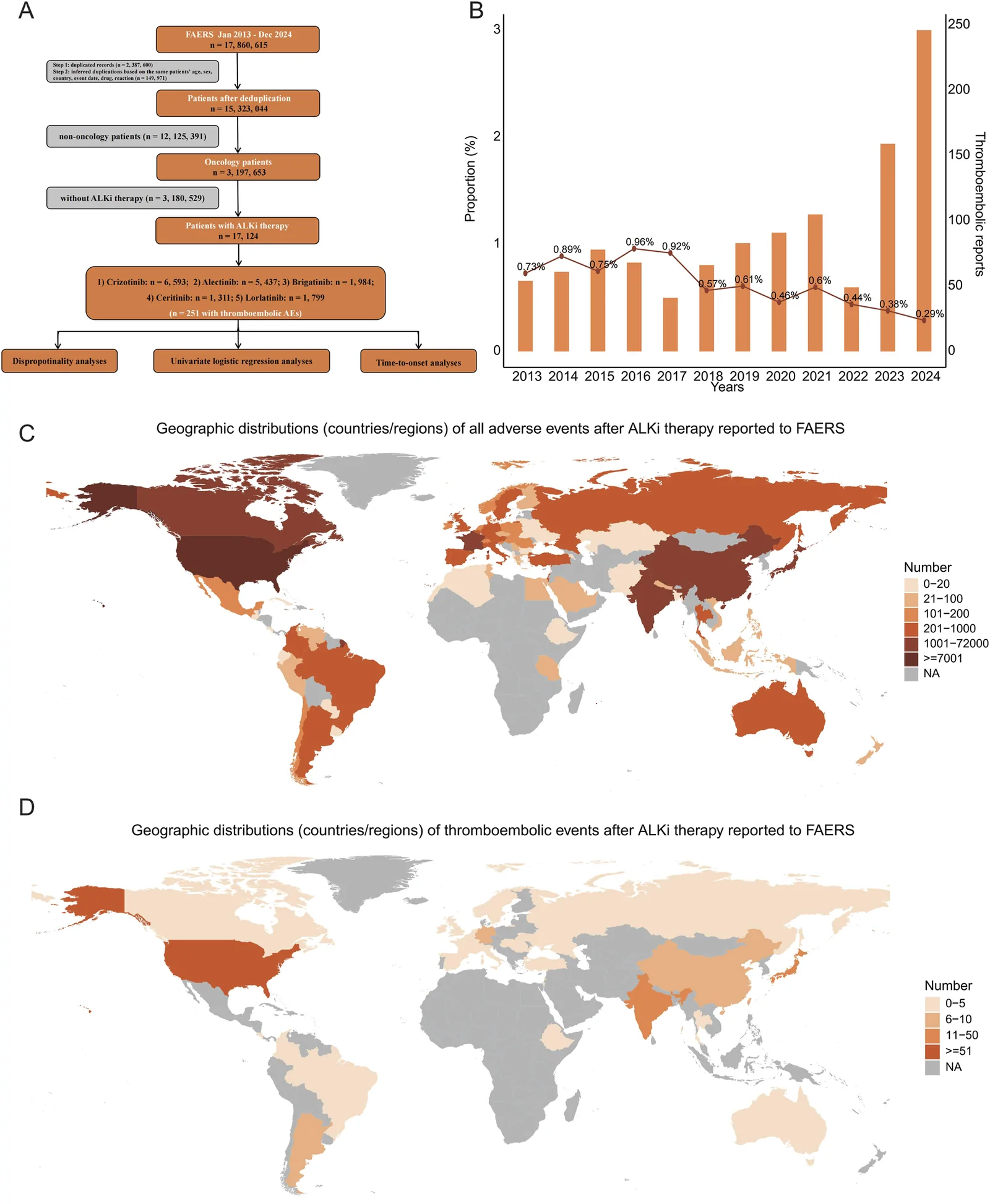
Data mining process and basic characteristics of adverse event (AE) reports following ALKi therapy. **(A)** Flow chart of the ALKi pharmacovigilance data mining process. Raw data were sourced from FAERS. The observational period was set from 2013 to 2024. **(B)** Chronological changes in total AE and throboembolic (TE) AE reports following ALKi therapy reported to FAERS from 2013 to 2024. Red bars represent the counts of TE reports deposited in FAERS, and gray lines delineate the proportion of TE events out of total AE reports annually. **(C,D)** Geographical distribution (countries/regions) of all AE reports and TE reports following ALKi therapy reported to FAERS.

After data deduplication (Figure 1A), there were 17, 124 patients experienced AEs after ALKi therapy from 2013 to 2024, among whom 251 reported TE. The annual proportion rate of TE out of the total AE reports following ALKi therapy were approximately 0.6%, albeit with slight fluctuation in the past decade (Figure 1B). AE reports were collected from 85 countries/regions, and the total number was 52, 808. Among which, there were 1,084 TE reports from 42 countries/regions (Figures 1C,D). Table 1 summarizes the basic demographics and other characteristics deposited in FAERS.

**TABLE 1:** Characteristics of adverse event reports for ALKi and non-ALKi users in the FAERS database.

​	ALKi		Non-ALKi		​
Age	n	%	n	%	P
<65 y	6, 945	(54.8)	1, 118, 338	(55.6)	0.75
≥65 y	5, 717	(45.2)	892, 823	(44.4)	
NA	1, 173, 830				​
Sex	n	%	n	%	P
Female	1, 454	(55.5)	533, 966	(59.9)	0.05
Male	1, 166	(44.5)	357, 377	(40.1)	
NA	2, 229, 749				​
TE reports	n	%	n	%	P
Y	251	(1.5)	51, 493	(1.6)	1
N	16, 832	(98.5)	3, 129, 077	(98.4)	
Outcomes	n	%	n	%	P
Death	3, 716	(21.8)	439, 138	(13.8)	1e-05
Non-fatal outcomes	13, 367	(78.2)	2, 741, 432	(86.2)	
Reporter occupation	n	%	n	%	P
MD	6, 591	(39.1)	815, 209	(26.4)	/
CN	6, 051	(35.9)	1, 245, 975	(40.3)	
OT	1, 553	(9.2)	480, 093	(15.5)	
PH	1, 476	(8.8)	286, 564	(9.3)	
Others	1, 174	(7.0)	264, 853	(8.5)	
NA	88, 114				

ALKi, anaplastic lymphoma kinase inhibitor; CN, consumer; TE, thromboembolic event; MD, physician; NA, not available; OT, other health-professional; PH, pharmacist.

A total of 456 TE events, each with a minimum individual count greater than three, were identified, of which venous events (67.5%) predominated over arterial events (1.1%), and 31.4% were classified as anatomically unspecified. Among these, 308 events (67.5%) were classified as venous TEs, including pulmonary embolism (n = 175), deep vein thrombosis (n = 90), pulmonary thrombosis (n = 22), pulmonary artery thrombosis (n = 6), venous thrombosis (n = 6), venous thrombosis limb (n = 6), and renal vein thrombosis (n = 3). In contrast, only five cases (1.1%) were identified as arterial TE, all of which were reported as cerebral thrombosis exclusively in the crizotinib group, and no arterial TE events were reported for the next-generation ALKi. The remaining 143 events (31.4%) were classified as anatomically unspecified (n of thrombosis = 113; n of embolism = 30) due to the lack of vascular attribution in the reporting terms.

**FIGURE 2:**
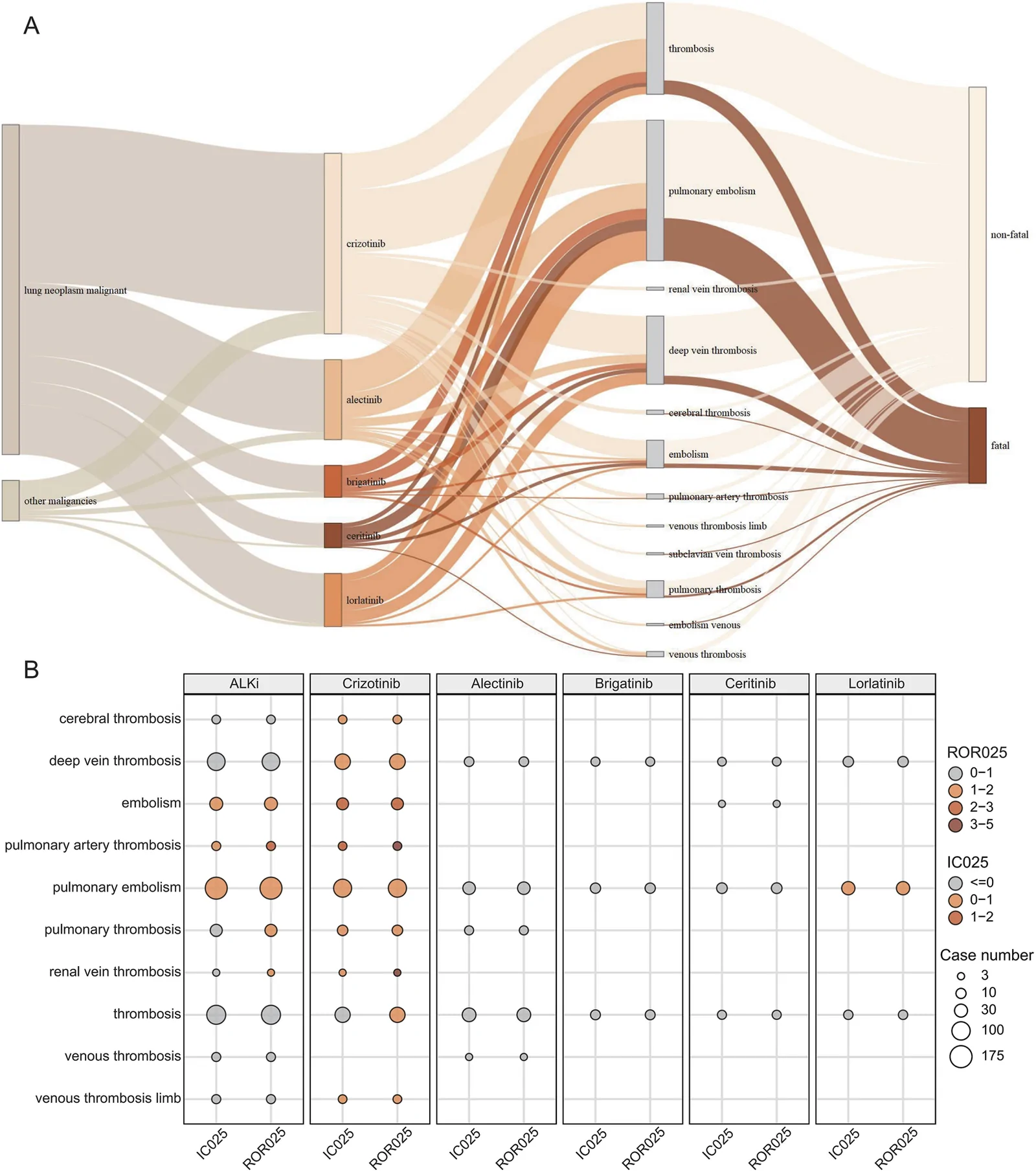
Characterization of PTs regarding TE following ALKi therapy in FAERS. **(A)** The evolutionary trajectory between diseases, ALKi subgroups, TE, and patients’ outcomes via the Sankey diagram in FAERS. The width of the flows represents the corresponding proportion of the nodes. **(B)** The overall and subgroup ALKis show PTs via heat map visualization in FAERS. The sizes of the circle correspond to the case numbers, while the gradient ramps from gray to red denote the ROR_025_ and IC_025_ values from small to large.

Approximately 90% of the patients receiving ALKis in these reported cases were treated for ALK-positive NSCLC. The most commonly used agent was crizotinib (50%), followed by alectinib (22%), lorlatinib (14%), brigatinib (9%), and ceritinib (5%). In total, there were 11 thromboembolic PTs screened out with a minimum case number of three, while pulmonary embolism, thrombosis, and deep vein thrombosis ranked the top three PTs linking to fatal outcomes (Figure 2A). Although there was no significant overall differences in TE rates between ALKi and non-ALKi users (see Table 1), the TE signal analysis revealed statistically significant associations between ALKi and pulmonary embolism (ROR_025_ = 1.20; IC0_25_ = 0.22), pulmonary artery thrombosis (ROR_025_ = 2.47; IC0_25_ = 0.60), and embolism (ROR_025_ = 1.58; IC0_25_ = 0.53), suggesting potential thromboembolic risks requiring clinical vigilance. Noteworthy, subgroup analysis further revealed that the statistically significant overall TE risk might be primarily driven by crizotinib, as evidenced by the positive safety signal strength of embolism (ROR_025_ = 2.66; IC0_25_ = 1.19), pulmonary artery thrombosis (ROR_025_ = 4.94; IC0_25_ = 1.01), pulmonary thrombosis (ROR_025_ = 1.29; IC0_25_ = 0.13), and limb venous thrombosis (ROR_025_ = 1.66; IC0_25_ = 0.08) observed only in the crizotinib subgroup. In contrast, alectinib, brigatinib, ceritinib, and lorlatinib did not reach the predefined positivity threshold (ROR_025_ > 1 and IC_025_ > 0), indicating that no disproportionate TE signal was detected for these agents in the real-world database. See Figure 2B.

**FIGURE 3:**
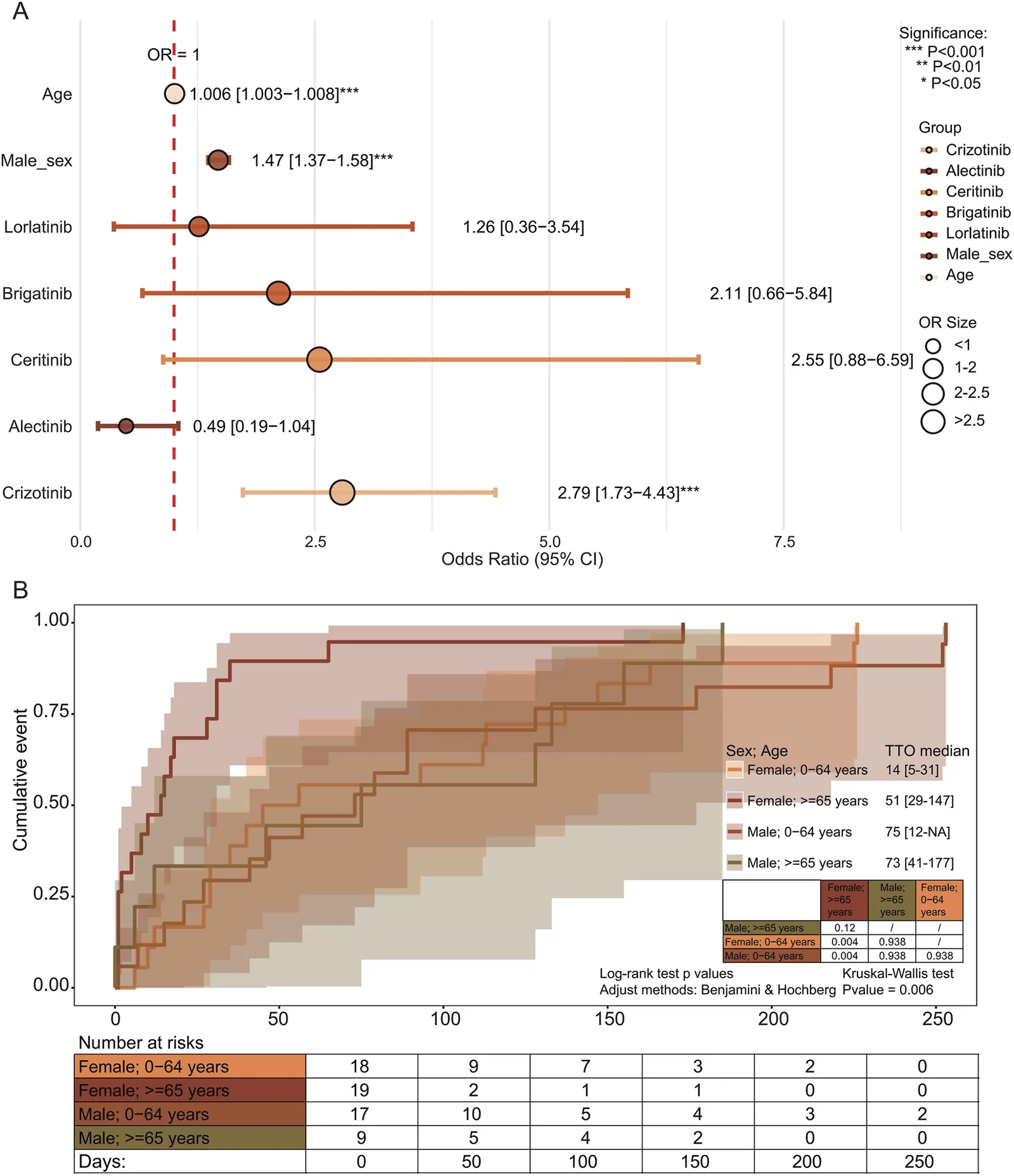
Exploratory relationship between risk factors and patients’ survival, and time-to-onset analysis of TE following ALKi therapy in FAERS. **(A)** The impact of ALKi subgroups on patients’ fatal outcomes following ALKi therapy, adjusting for demographics, via forest plot visualization in both FAERS. The sizes of the circle and the error bars correspond to the odds ratio and its 95% confidence interval. **(B)** The cumulative distribution curve of the onset time of TE following ALKi therapy in FAERS.

Multivariate logistic regression analysis has further identified that age (OR [95% CI] = 1.006 [1.003–1.008]; P = 2.40e-06), male sex (1.47 [1.37–1.58]; P = 2.00e-16), and crizotinib (2.79 [1.73–4.43]; P = 1.59e-05) were statistically significant contributors to fatal outcomes in TE cases (Figure 3A). Among patients who developed TE within the first year, the time-to-TE onset did not significantly differ between those receiving ALKi therapy and those not receiving ALKi therapy (49 versus 56.5 days; P = 0.46), nor between female or male patients (44 versus 53 days; P = 0.46). Notably, female ALKi users under 65 years old (n = 18) reported significantly earlier TTO of TE (14 days [5–31]; P_adjusted_ = 0.006) than other groups treated with ALKi (Figure 3B).

## Discussion

For alectinib, brigatinib, ceritinib, and lorlatinib, the ROR_025_ and IC_025_ values did not reach the predefined positivity threshold, indicating that no disproportionate TE reporting signal was detected for these agents in the FAERS database. This observation is consistent with the relatively neutral TE profiles reported in prior meta-analysis (Gourzoulidis et al., 2025), although it should not be over-interpreted as a quantitative comparative advantage, given the inherent limitations of the spontaneous reporting data. The markedly elevated TE signal observed with crizotinib contrasts with the relatively neutral TE profiles of the next-generation ALKi, a disparity potentially attributable to differences in kinase selectivity and off-target effects on platelet–endothelial homeostasis. As the first-generation ALKi, crizotinib exhibits multi-kinase inhibitory activity, targeting MET and ROS1 (FDA, 2011; Sahu et al., 2013), kinases implicated in endothelial integrity (Trusolino et al., 2010) and platelet function (Pietrapiana et al., 2005). In contrast, the next-generation ALKi demonstrates enhanced ALK specificity, which may reduce collateral prothrombotic effects. Furthermore, the interplay between MET and vascular endothelial growth factor (VEGF) signaling in tumor angiogenesis (Zhang et al., 2016) increases the possibility that crizotinib’s MET inhibition could indirectly perturb the VEGF receptor (VEGFR) pathway, which is known to influence thrombosis (Touyz et al., 2018). However, these mechanistic hypotheses remain speculative, underscoring the need for functional studies to delineate the TE risk differentials between crizotinib and the newer ALKi.

Our stratified inventory reveals a clear predominance of venous over arterial TE in ALKi-treated patients. The positive TE disproportionality signal was predominantly attributable to crizotinib, and this pattern consistently held across VTEs. Notably, the only ATE captured, excluding those with unspecified anatomy, was reported exclusively in the crizotinib group, further underscoring its distinct prothrombotic profile. However, the precise pathophysiological mechanisms underlying these rare ATEs remain elusive from the present data, and it remains unclear whether they share common etiology with crizotinib-associated VTEs. Our findings align with broader evidence from diverse study designs. First, it is important to recognize that ALK-positive NSCLC patients inherently carry an elevated baseline TE risk. A population-based cohort study demonstrated that even before or independent of treatment, ALK-positive patients had an 87% higher risk of VTEs than ALK-negative patients, with a 12-month incidence of 17.7% (Icht et al., 2023). Building upon this high-risk baseline, treatment with crizotinib appears to further exacerbate the risk. A single-institution cohort found that the VTE risk was significantly elevated during active treatment with crizotinib (HR = 8.72), and VTE occurrence was associated with a 3.5-fold increased mortality risk (Zaborowska-Szmit et al., 2024). The pediatric population is not spared, with 20% of children receiving crizotinib-containing regimens experiencing TEs in the ANHL12P1 trial (Lowe et al., 2023). Notably, head-to-head safety comparisons between individual ALKi are scarce in clinical trial designs; however, a Bayesian network meta-analysis, which included eight RCTs, found that crizotinib was associated with a significantly higher TE risk than chemotherapy (OR = 0.28), brigatinib (OR = 0.31), and ceritinib (OR = 0.13), ranking the highest in TE risk among all treatment regimens (SUCRA = 0.99) (Qi et al., 2024). These diverse lines of evidence consistently support our real-world pharmacovigilance observations and reinforce the clinical imperative for TE risk stratification in crizotinib-treated patients. As this study suggests that VTE is the dominant clinical presentation of ALKi-associated TE safety signals, from a practical standpoint, VTE prophylaxis and surveillance (e.g., anticoagulation awareness) could be prioritized, particularly in patients receiving crizotinib. We also acknowledge that a substantial proportion of cases (31.4%) were captured under the non-specific terms “thrombosis” and “embolism,” which lack anatomical attribution, representing an inherent limitation of MedDRA PT terminology. Additionally, detailed vascular localization information is not available in the FAERS database, preventing definitive subtype assignment at the individual case level.

The clinical relevance of characterizing crizotinib’s TE risk extends beyond its current market share. Gourzoulidis et al., (2025) reported a practice shift from crizotinib to alectinib among ALK-positive NSCLC patients in Greece, driven by the superior efficacy and favorable safety profiles of the next-generation ALKis, consistent with evolving clinical guidelines. Nevertheless, our findings remain critically important in healthcare systems with limited access to newer agents, whether due to reimbursement constraints or regional availability. In such settings, crizotinib continues to be prescribed, and clinicians need to be fully informed of its real-world TE safety profile to guide risk–benefit assessments and implement appropriate safety monitoring in routine practice. By providing granular, drug-specific TE risk estimates derived from a globally representative real-world population, our study equips clinicians with actionable evidence to support clinical decision-making and optimize patient safety when crizotinib remains the only or most accessible therapeutic option.

The association of older age and male sex with fatal TE outcomes aligns with population-level data on cancer-associated thrombosis (Fernandes et al., 2019). Notably, the older age with fatal TE outcomes, while statistically significant, warrants careful clinical interpretation. The estimated per-year increase is modest, suggesting that age alone should not be considered a dominant driver of TE-related mortality. Rather, its clinical relevance lies in its cumulative effect over time and its interaction with other risk factors; male sex and crizotinib use are independently associated with elevated risk, and the combined effect may yield a substantially higher absolute risk profile. The median TE onset within 50 days of ALKi initiation underscores the need for heightened vigilance during early treatment. For high-risk patients (e.g., male patients >65 years receiving crizotinib), proactive thromboprophylaxis guided by validated risk scores [e.g., Khorana score (Mulder et al., 2019)] may be warranted. The comparatively lower thrombotic risk profile of the next-generation ALKi supports their preferential use as a safer therapeutic option for patients with baseline TE predispositions. Although, the observed earlier TE onset in younger female ALKi recipients (<65 years) introduces clinical complexity, this phenomenon may be attributed to hormone-driven mechanisms or genetic thrombophilias, potentially exacerbated by ALKi-mediated endothelial dysfunction. Nevertheless, the current evidence remains insufficient to distinguish true biological causality from statistical artifacts, necessitating prospective studies with dedicated subgroup analyses and mechanistic investigations into sex-specific ALKi pharmacodynamics.

As with observational pharmacovigilance studies utilizing FAERS data, several inherent limitations warrant cautious interpretation. First and foremost, the FAERS is a spontaneous reporting system that lacks exposure denominators; therefore, disproportionality measures cannot be interpreted as incidence rates, cumulative risks, or valid comparative effect estimates between drugs. Reporting biases, including under-reporting, selective reporting, and differential reporting driven by media attention or regulatory actions, cannot be quantified or fully adjusted for. As a supportive observation, the annual TE proportion among ALKi-related reports remained stable at approximately 0.6% over 12 years, suggesting no major temporal reporting distortions. To this end, our analyses were strictly hypothesis-generating or hypothesis-substantiating and served to complement, rather than replace, causal evidence from prospective clinical trials or well-designed cohort studies with defined denominators. Second, the absence of granular clinical parameters, such as tumor burden, anticoagulant adherence, and performance status, prevents comprehensive adjustment for residual confounding from treatment-related hypercoagulability or immobilization. This is particularly relevant when comparing crizotinib with next-generation ALKis as these agents were used in different treatment eras and patient populations that secular trends in clinical practice, evolving standards of care, and changes in thromboprophylaxis protocols may all contribute to the observed differences in TE signals. Although approximately 90% of the ALKi-treated patients in our dataset had NSCLC, which reduces disease-related heterogeneity, we cannot rule out confounding by indication subtypes or time-dependent factors. Third, survival outcomes may be confounded by institutional variations in thromboprophylaxis protocols rather than reflecting direct ALKi therapeutic effects. These methodological constraints highlight the necessity of validating the current findings through prospective registries with protocolized data collection and adjudicated endpoint ascertainment. Last but not the least, the exploratory subgroup TTO analysis stratified by sex and age was based on small event counts (as shown in Figure 3B, with baseline n ranging from 9 to 19 per subgroup), limiting statistical power and precision. Although the observed difference remained significant after Benjamini–Hochberg correction for multiple comparisons, the small sample size precludes definitive conclusions, and we cannot exclude the possibility of a false-positive finding. Accordingly, these results should be considered exploratory and hypothesis-generating rather than confirmatory. Independent validations are warranted in future prospective studies.

Future prospective cohort studies incorporating longitudinal monitoring of thrombosis-related biomarkers are warranted to elucidate causal relationships between ALKi pharmacodynamics and TE. Furthermore, developing machine learning-driven risk stratification models that integrate multi-omics profiles, tumor-specific hypercoagulability states, and treatment parameters could enable precision antithrombotic prophylaxis in oncological practice.

## Conclusion

In conclusion, this study highlights the heterogeneous TE risks across ALKi, emphasizing the importance of agent-specific risk–benefit assessments. Although the next-generation ALKis offer improved TE safety profiles, vigilant monitoring and tailored interventions remain essential, particularly for crizotinib-treated patients with predisposing risk factors. Notably, the TE risk associated with ALKi is overwhelmingly venous in nature, supporting a prioritized focus on VTE management in clinical practice.

## Data Availability

Publicly available datasets were analyzed in this study. This data can be found here: https://fis.fda.gov/extensions/FPD-QDE-FAERS/FPD-QDE-FAERS.html.
